# RAGE Silencing Ameliorates Neuroinflammation by Inhibition of p38-NF-κB Signaling Pathway in Mouse Model of Parkinson’s Disease

**DOI:** 10.3389/fnins.2020.00353

**Published:** 2020-04-29

**Authors:** Xiaoli Wang, Xiaoxuan Sun, Mengyue Niu, Xiaona Zhang, Jing Wang, Chang Zhou, Anmu Xie

**Affiliations:** Department of Neurology, The Affiliated Hospital of Qingdao University, Qingdao, China

**Keywords:** PD, neuroinflammation, RAGE, NF-κB, p38MAPK, COX-2

## Abstract

Accumulating evidence suggested that neuroinflammation played a crucial role in dopaminergic neuronal death in Parkinson’s disease (PD). The receptor for advanced glycation end products (RAGE), a multi-ligand receptor of the immunoglobulin superfamily, has been proposed as a key molecule in the onset and sustainment of the inflammatory response. Engagement of RAGE contributed to neuroinflammation by upregulating nuclear factor-κB (NF-κB) as well as cytokines. The aim of the present study was to investigate the expression of RAGE in 1-methyl-4-phenyl-1,2,3,6-tetrahydropyridine (MPTP)-treated mice and elucidate the RAGE signal pathway involved in the inflammation. Results showed that RAGE protein and pro-inflammatory cytokines cyclooxygenase type 2 (COX-2) were upregulated in MPTP-treated mice. Further experiments showed that RAGE ablation inhibited phosphorylation of IκB and p38 and protected nigral dopaminergic neurons against cell death in the substantia nigra (SN). These results suggested that RAGE participated in the pathogenesis of PD by neuroinflammation and p38MAPK-NFκB signal pathway may be involved in the process. Moreover, interfering with RAGE signaling pathway may be a reasonable therapeutic option in slowing PD development and progression.

## Introduction

Parkinson’s disease (PD) is the second most common neurodegenerative disorder characterized by progressive loss of dopaminergic neurons and accumulation of aggregated α-synuclein in the substantia nigra (SN). PD seems to result from a complicated interplay of genetic and environmental factors affecting numerous fundamental cellular processes (Lees et al., 2009; Kalia and Lang, 2015). However, the pathogenic mechanisms that ultimately cause PD are still unclear. Increasing evidence has demonstrated that neuroinflammatory mechanisms might contribute to the cascade of events leading to neuronal degeneration (Qian et al., 2010; Herrero et al., 2015). Both cerebrospinal fluid and post-mortem brain were investigated and found accumulation of pro-inflammatory cytokines, including cyclooxygenase type 2 (COX-2), tumor necrosis factor-α (TNF-α), interleukin-6 (IL-6), and interleukin-1β (IL-1β) (Nagatsu et al., 2000; Frank-Cannon et al., 2009). Moreover, activated microglia was recently reported in the SN and putamen of PD patients visualized by positron emission tomography (Iannaccone et al., 2013). These findings shed light on promising therapeutic strategies aimed at downregulating these inflammatory processes to slow the progression of PD.

The receptor for advanced glycation end products (RAGE) is a multiligand protein belonging to the immunoglobulin superfamily that can bind with a broad range of ligands such as advanced glycation end products (AGEs), amyloid β (Aβ), high mobility group box 1 (HMGB1), and S100/calgranulin (Schmidt et al., 2001; Ding and Keller, 2005). Ligation of RAGE triggers a series of cellular signaling events, including the activation of transcription factor nuclear factor-κB (NF-κB), leading to the production of pro-inflammatory cytokines, and causing inflammation. The stimulation of RAGE is able to activate the mitogen-activated protein kinase (MAPK) signaling cascades, which thereby release and activate NF-κB in the downstream (Tobon-Velasco et al., 2014). Indeed, RAGE involvement has been demonstrated in the pathogenesis of a number of inflammation-associated conditions including diabetes, atherosclerosis, arthritis, and Alzheimer’s disease (AD) (Chuah et al., 2013). Evidence shows that RAGE were highly expressed in PD patients when compared to age-matched controls and RAGE gene polymorphisms were associated with sporadic PD in Asians (Guerrero et al., 2013; Gao et al., 2014), suggesting that RAGE might play a crucial role in the pathogenesis of PD. Recent studies showed S100B and HMGB1 were increased in PD and ablation protected against 1-methyl-4-phenyl-1,2,3,6-tetrahydropyridine (MPTP)-induced toxicity through the RAGE signal pathway (Sathe et al., 2012; Santoro et al., 2016). In addition, RAGE-deficient mice treated with MPTP showed more surviving dopaminergic neurons than their control littermates. Also, primary dopaminergic neurons from RAGE-deficient mice were partially protected against MPP + induced cell death. Therefore, we speculated that RAGE may contribute to the chronic inflammation and progressive dopaminergic neurodegeneration in PD models through NF-κB activation. Blockage of the RAGE may provide new therapeutic avenues for the disease.

MPTP is a common neurotoxin for inducing PD models in mice and chronic administration of MPTP induced the formation of nigral inclusions, which is similar to those observed in idiopathic PD (Blandini and Armentero, 2012). In this study, we aim to confirm the upregulation of RAGE in MPTP-treated mice and elucidate the role of RAGE in neuroinflammation and underlying mechanisms by silencing RAGE with lentivirus transfection.

## Materials and Methods

### Reagents

Unless otherwise indicated, all the antibodies in this experiment were purchased from Cell Signal Technology (Hertfordshire, England). Primary antibodies against RAGE and COX2 were supplied by Abcam (Cambridge, United Kingdom) and secondary antibody against rabbit IgG was supplied by Santa Cruz Biotechnology (Santa Cruz, CA, United States). Polyclonal antibodies tyrosine hydroxylase (TH) were purchased from Millipore (Bedford, MA, United States) and Alexa Fluor^®^ 555-conjugated secondary goat anti-rabbit antibody was purchased from Life Technologies (United States). Lentivirus small interference RAGE and the negative control lentiviral vectors were purchased from Shanghai GeneChem (Shanghai, China). MPTP⋅HCl was purchased from Sigma Chemical (St. Louis, MO, United States). MPTP⋅HCl was dissolved in physiological saline (0.9%) at a concentration of 6.0 mg/ml.

### Animals and Groups

Male C57BL/6 mice (25–30 g of body weight) were obtained from Beijing Vital River Laboratory Animal Technology. The animals were kept in cages under 20 ± 2°C with a 12/12-h light–dark cycle and free access to water and food. In total, 78 mice were randomly divided into six groups: (1) control group, (2) MPTP group, (3) negative control of lentiviral vectors of RAGE (RAGE-NC) group, (4) lentivirus small interference RAGE (RAGE-siRNA) group, (5) MPTP + RAGE-NC group, and (6) MPTP + RAGE-siRNA group. In each group, 13 mice were included. The mice in MPTP and normal control groups were given two intraperitoneal injections of MPTP (30 mg/kg) at 2-day intervals per week for five consecutive weeks (Petroske et al., 2001; Meredith et al., 2008) or a comparable volume of 0.9% saline, respectively, while the animals in RAGE-NC and RAGE-siRNA groups were transfected with lentivirus by stereotaxic surgery in the brain accordingly. The mice in the MPTP + RAGE-NC group and the MPTP + RAGE-siRNA group were treated with MPTP twice a week for five consecutive weeks, 1 week after the transfection of appropriate lentivirus. At the seventh week, a rotarod test was conducted before harvesting brain tissues of mice.

### Stereotaxic Surgery and Lentivirus Transfection

Mice were anesthetized using isoflurane (3% induction, 1.5% maintenance) and then fixed in a stereotaxic frame (RWD Life Science Co. Ltd.). The skin of the surgical site was opened to reveal the skull, and the bregma was determined. Then, the skull was delicately bored with a dental drill at a location just above the SN. Bilateral injections were made at the following coordinates: 3 mm posterior to the bregma, 1.3 mm lateral to the midline, and 4.7 mm ventral from the dura (Paxions and Franklin, 2019). One-microliter syringe with 26-gauge blunt tip needle (Hamilton) was slowly lowered into the brain parenchyma and 0.3 μl of lentiviral preparation (3 × 10^8^TU/ml) was injected over a period of 5 min. The needle was left in place for an additional 5 min to prevent reflux and then slowly removed. The hole in the skull was filled with sterile bone wax, and the skin was replaced and closed using surgical staples. Mice were kept on a heating pad until recovery from anesthesia and returned to their home cages.

### Rotarod Test

A rotarod (Med Associates, Inc.) test was performed to measure motor balance and coordination. The velocity of the rod (diameter, 5 cm) was from 4 to 40 r/min. Before starting the experiment, a rotarod train was performed for three consecutive days at a fixed speed (5 r/min). Latency until fall was automatically recorded by magnetic trip plates. Mice were given a maximum cutoff latency of 300 s to avoid stress and fatigue. For each animal, the experiment was repeated three times and the average time was calculated.

### Determination of Dopamine (DA) and Its Metabolites

The contents of dopamine (DA) and its metabolites 3,4-dihydroxyphenylacetic acid (DOPAC) and homovanillic acid (HVA) in the striatum were subjected to high-pressure liquid chromatography coupled with electrochemical detection (HPLC-ECD, Waters Corp., Milford, MA, United States) and expressed as a concentration in nanograms (ng) per milligram (mg) of protein. Briefly, animals were sacrificed and tissue samples were dissected at a temperature of 4°C. The striatum was weighed and homogenized in 100 μl of ice-cold liquid A (0.4 M perchloric acid) and centrifuged at 12,000 r/min for 20 min at 4°C. After centrifugation, 80 μl of the supernatant mixed with 40 μl of ice-cold liquid B (20 mM citromalic acid-potassium, 300 mM dipotassium phosphate, 2 mM EDTA-2Na) was then centrifuged at 12,000 r/min for 20 min at 4°C. After centrifugation, the supernatants were frozen at −80°C or immediately applied to the HPLC system. Dialysis samples were assayed for DA, DOPAC, and HVA by HPLC with electrochemical detection.

### Western Blot Analysis

Sample tissues (dissected the SN) were lyzed with lysis buffer containing RIPA Lysis Buffer and PMSF (99:1). The mixture was centrifuged at 12,000 r/min for 20 min at 4°C and protein concentrations in the supernatant were determined by BCA colorimetric protein assay kit (Beyotime, Jiangsu, China). A total of 20 μg of protein was separated by 10% SDS-polyacrylamide gels and then transferred to polyvinylidene fluoride membranes (Immobilin-P, Millipore Corp., MA, United States). After 2 h blocking with 10% non-fat milk at room temperature, membranes were incubated overnight with primary antibody of rabbit phospho-p38 (1:2000), p38 (1:2000), phospho-IκB (1:1000), IκB (1:1000), RAGE (1:2000), TH (1:4000), COX-2 (1:2000), and β-actin (1:10,000). After washing, the membranes were incubated with anti-rabbit secondary antibodies conjugated to horseradish peroxidase (1:10,000) for 2 h at room temperature. The antigen–antibody complexes were detected with enhanced chemiluminescence reagent and visualized by Imager (UVP Biospectrum 810, United States).

### TH Fluorescence Immunohistochemistry and Detection of Infected Cells

The mice were transcardially perfused with normal saline and 4% paraformaldehyde in 0.1 M phosphate buffered saline (PBS, pH 7.4) after anesthesia. Brains were dissected and post-fixed in 4% paraformaldehyde for 24 h followed by 30% sucrose at 4°C for 48 h. Twenty-micrometer coronal sections were cut through the entire SN on a freezing cryostat (Leica, Germany) and the sections were placed on a tissue culture plate in PBS. Free-floating sections were processed for TH immunohistochemistry. Briefly, sections were incubated overnight with a rabbit anti-mouse monoclonal TH (1:4000) in PBS at 4°C, followed by a 2-h incubation with Alexa Fluor^®^ 555-conjugated secondary goat anti-rabbit antibody (1:1000) for 2 h at room temperature. Then, fluorescent signals were visualized by Axiophot fluorescence microscope (Zeiss, Oberkochen, Germany) and the number of stained TH neurons in SN on each section was counted at a magnification of 100 × by Image Pro Plus software. Estimation of the total number of TH neurons per brain was obtained by multiplying the counts by six sections.

The vector of siRAGE carrying the green fluorescent protein (GFP) gene was a tracing carrier for siRNA expression. The fluorescent signals were also visualized by axiophot fluorescence microscope and merged with the TH staining neurons (red fluorescent) to observe the distribution of infection.

### Statistical Analysis

One-way analysis of variance (ANOVA) followed by Student–Newman–Keuls and unpaired *t*-test were performed to show significance of differences between group means. Data were normally distributed between the groups. All data were expressed as mean values ± standard error of the mean. Values of *p* < 0.05 were considered significant.

## Results

### RAGE Suppression Improved the Motor Deficits of MPTP Mice

**FIGURE 1:**
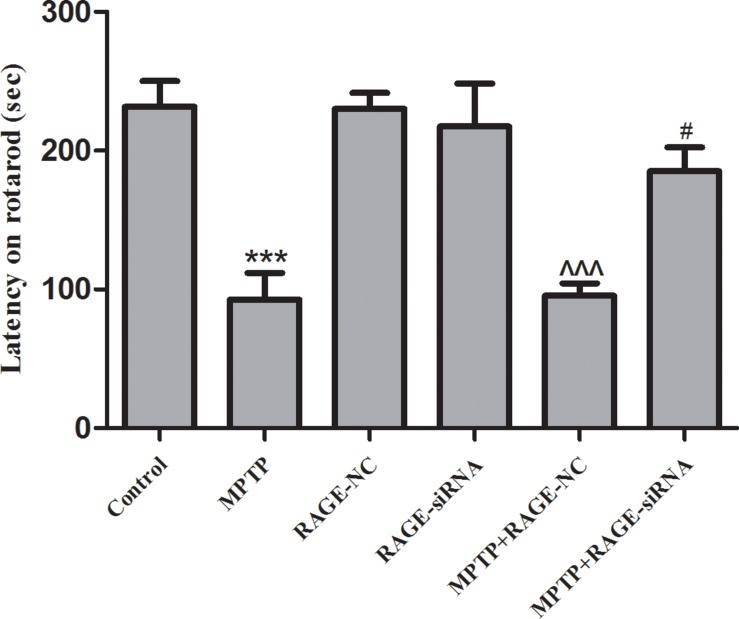
The rotarod of mice. The latency time of MPTP-treated mice was significantly reduced compared to that of controls. After RAGE siRNA administration in MPTP + RAGE-siRNA group, the motor deficits of mice partly improved compared with that of MPTP + RAGE-NC mice. There was no statistical difference of latency time between control group and RAGE-NC group (mean ± SEM, *n* = 10, ***p < 0.001, compared with control; ^^^*p* < 0.001, compared with RAGE-NC group; #*p* < 0.05, compared with MPTP + RAGE-NC).

Intraperitoneal injections of MPTP in five consecutive weeks lead to chronic lesions of the nigrostriatal dopaminergic pathway. Rotarod test is regarded as a quantitative index of SN lesion severity. The rotarod of all groups is shown in Figure 1. The time for which MPTP-treated mice remained on the beam was significantly reduced (92.5 ± 19.5s vs. 231.5 ± 18.5 s, *p* < 0.001) compared to controls. The latency time in the RAGE-NC group and RAGE-siRNA group had no difference compared to the control group, respectively. Further studies showed that the motor deficits of mice partly improved in the MPTP + RAGE siRNA when compared with the MPTP + RAGE-NC group (185.1 ± 17.3s vs. 95.29 ± 8.9, *p* < 0.05). Thus, we demonstrated that the inhibition of RAGE alleviated MPTP-induced motor balance and coordination deficit.

### RAGE Protein Levels Were Increased in Substantia Nigra Pars Compacta of MPTP-Treated Mice and Was Suppressed by siRNA Targeting RAGE

**FIGURE 2:**
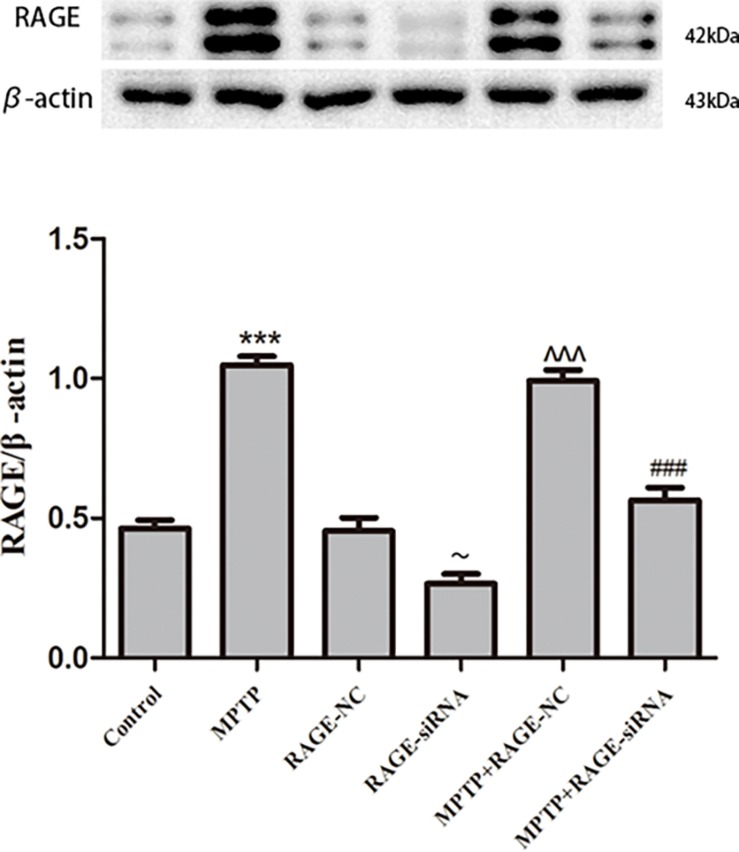
RAGE protein level in substantia nigra pars compacta. RAGE was increased in MPTP-treated mice compared with that of control group. In the MPTP + RAGE-siRNA group, this effect was partly inhibited by treatment with RAGE siRNA. RAGE protein level in control had no difference compared with RAGE-NC group, indicating lentivirus administration alone showed no effect on RAGE expression. While RAGE protein level in RAGE-siRNA group decreased respect to the RAGE-NC group, indicating lentivirus small interference RAGE affected RAGE expression successfully (mean ± SEM, *n* = 5–8, ***p < 0.001, compared with control group; ∼*p* < 0.05, compared with RAGE-NC; ^^^*p* < 0.001, compared with RAGE-NC group; ###*p* < 0.001, compared with MPTP + RAGE-NC group).

To investigate the expression alterations of RAGE, we detected the expression levels of RAGE by Western blot in MPTP-treated mice. Western blot showed a significant upregulation of RAGE expression after 5 weeks of MPTP treatment compared to the normal control group. RAGE protein level in RAGE-siRNA-treated group decreased with respect to the RAGE-NC group (*p* < 0.05), which meant that lentivirus small interference RAGE affected RAGE expression successfully. Furthermore, there was no statistical difference in RAGE protein expression between control group and RAGE-NC group, which indicated that lentivirus vector administration alone had no effect on RAGE expression. However, compared with the MPTP + RAGE-NC group, decreased RAGE protein levels were detected in MPTP + RAGE-siRNA treated mice (Figure 2). Thus, MPTP treatment resulted in elevated RAGE level, and RAGE siRNA can successfully interfere with RAGE mRNA transcription and then inhibited its protein expression.

### RAGE Suppression Increased Levels of DA, DOPAC, and HVA in Striatum

**FIGURE 3:**
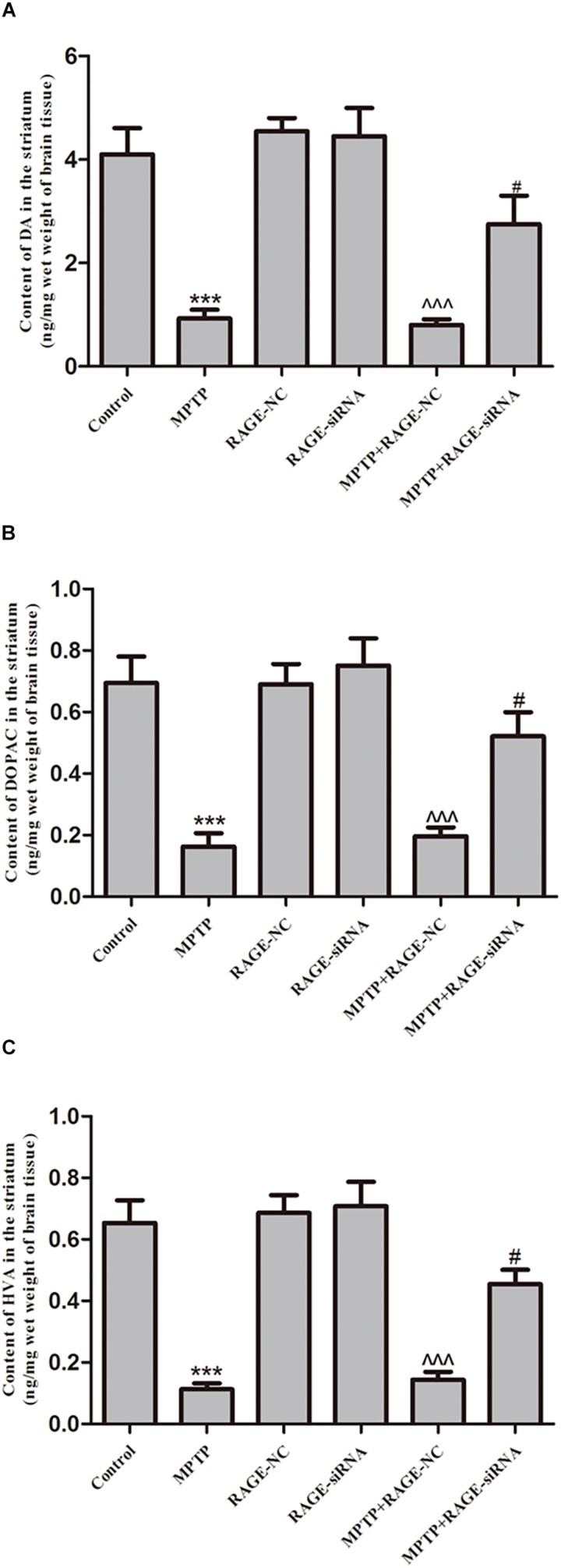
The content of dopamine and its metabolites DOPAC and HVA in striatum. MPTP treatment caused the reduction of **(A)** DA, **(B)** DOPAC, and **(C)** HVA. Before MPTP injection, mice were treated with RAGE siRNA, which partly reversed MPTP-induced dopamine depletion (mean ± SEM, *n* = 7–8, ***p < 0.001, compared with control; ^^^*p* < 0.001, compared with RAGE-NC group; #*p* < 0.05, compared with MPTP + RAGE-NC group).

Dopamine and its metabolites DOPAC and HVA levels in the striatum were determined by HPLC apparatus. The mean levels of DA, DOPAC, and HVA in the striatum of PD group were significantly decreased compared with control group. The DA and its metabolite levels of mice treated with MPTP + siRNA-NC were not obviously different with those in MPTP-injected animals. However, after MPTP administration, RAGE siRNA partly reversed MPTP-induced DA depletion in MPTP + RAGE-siRNA group (Figure 3).

### RAGE Suppression Protected DA Neurons and Mitigates MPTP-Induced Neurodegeneration

**FIGURE 4:**
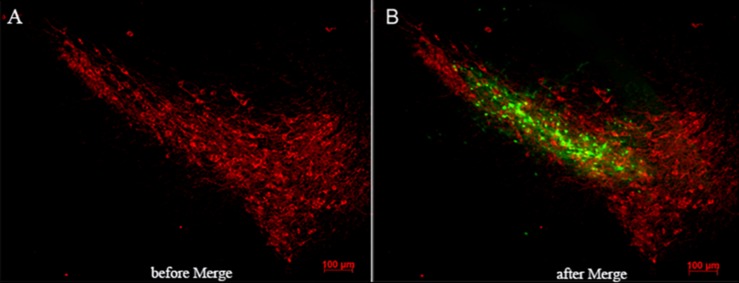
Lentivirus was injected in the right location (substantia nigra). **(A)** Immunofluorescence of TH-positive neurons (red-labeled) in substantia nigra. **(B)** Merged image by RAGE-siRAGE lentivirus vectors (green-labeled) and TH neurons in substantia nigra (magnification: 100×).

The dopaminergic neurons in SN were marked by TH antibody using immunofluorescence and tracked by expressing red fluorescent protein. RAGE-NC or RAGE-siRAGE lentivirus vectors contained GFP gene and were tracked by expressing GFP at the transfected localization. GFP-positive cells were observed along the needle track and SN. Double labeling in neurons showed lentivirus-infected TH neurons and other neuron cells in SN (Figure 4).

**FIGURE 5:**
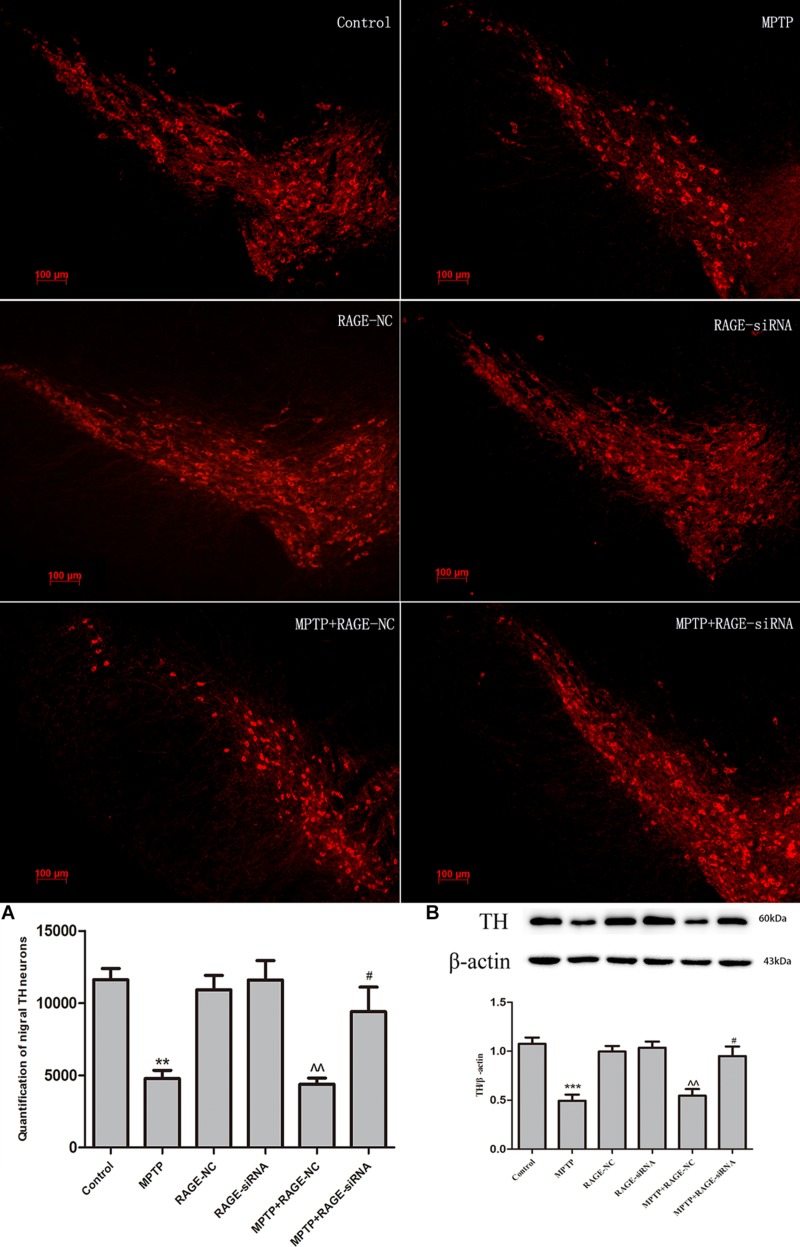
Dopaminergic neurons staining and estimation of the total number of TH neurons in substantia nigra in six groups. MPTP administration resulted in severe nigrastriatal lesions with a marked loss of TH neurons (MPTP group vs. control). Interestingly, RAGE-siRNA treatment partly increased the survival of TH neurons (MPTP + siRNA group vs. MPTP + RNA-NC group). RAGE-NC treatment alone did not produce any effect on the dopaminergic neuronal survival ratio compared to the control group (RAGE-NC group vs. control). The quantification of TH neurons and TH protein levels in SN were lined with these changes **(A,B)** (mean ± SEM, *n* = 5 ***p < 0.001, compared with control; ^^*p* < 0.001, compared with RAGE-NC group; #*p* < 0.05, compared with MPTP + RAGE-NC group).

In light of the MPTP-induced upregulation of RAGE, we further investigated whether this receptor was involved in the nigrostriatal degeneration. Stereological counts of SN dopaminergic neurons were defined by TH staining. MPTP administration resulted in severe nigrastriatal lesions with a marked loss of approximately 61.3% of TH neurons in the SN compared with the control group. We further demonstrated whether RAGE suppression could promote DA neurons’ survival. Interestingly, RAGE-siRNA treatment partly increased the survival of TH neurons and the survival ratio was 43.2% higher than the MPTP + RAGE-NC-treated mice. There was no significant difference in TH neurons between MPTP + RAGE-NC group and MPTP group. RAGE-NC treatment alone did not produce any effect on the dopaminergic neuronal survival ratio compared to the control group (Figure 5).

Immunoblotting results demonstrated that MPTP injection significantly decreased the protein expression of TH in the SN. While RAGE-siRNA treatment significantly antagonized the MPTP-induced decrease in TH protein level in SN (Figure 5).

### RAGE Suppression Reduced the Expression of COX2 and Inhibited NF-κB Activation, Ameliorating RAGE-Induced Inflammation

**FIGURE 6:**
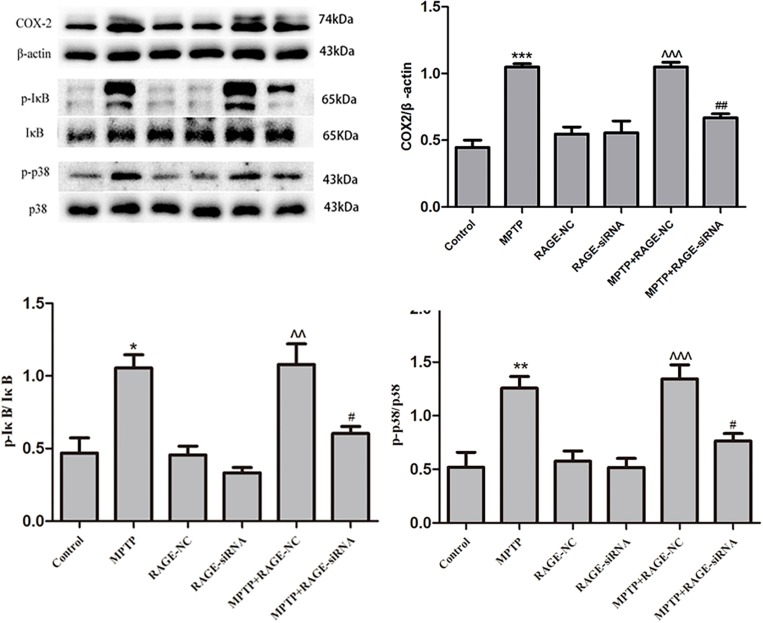
Neuroinflammation induced by RAGE activation was involved in the phosphorylation of IκB and p38. COX2 expression was increased after MPTP injection, accompanied by the elevated level of phosphorylation of IκB and p38. This effect was partly inhibited by treatment with RAGE siRNA. In RAGE-NC, COX2, p-IκB, and p-p38 expressions were not significantly changed compared to control group (mean ± SEM, *n* = 5, ***p < 0.001 or **p < 0.01 or *p < 0.05, compared with control; ^^^*p* < 0.001 or ^^*p* < 0.01, compared with RAGE-NC group; ##*p* < 0.01 or #*p* < 0.05, compared with MPTP + RAGE NC group).

Ligands binding to RAGE stimulates COX-2 expression, which is involved in pathogenesis of brain inflammation. Activation of NF-κB is known to mediate MPTP-induced inflammatory gene expressions. IκB phosphorylation induces the dissociation of NF-κB followed by translocation of NF-κB into the nucleus and gene transcription of pro-inflammatory cytokines including COX2. Therefore, we measured the levels of pro-inflammatory cytokines and phosphorylated IκB by Western blot. Compared with the control group, MPTP treatment induced a significant increase of COX2 and phosphorylated IκB. Moreover, treatment with RAGE-siRNA partly decreased the levels of COX2 and phosphorylated IκB in MPTP + RAGE-siRAGE group, respectively. Meanwhile, the levels of COX2 and phosphorylated IκB were not significantly different among RAGE-NC group, RAGE-siRNA group, and control group. This demonstrated that RAGE-siRNA reduced the expression of COX2 and phosphorylated IκB, suggesting that RAGE suppression could ameliorate inflammation (Figure 6).

### RAGE Suppression Inhibited p38 Phosphorylation

The MAPK signal pathways especially p38 MAPK plays an important role in controlling the NF-κB activation and pro-inflammatory cytokine synthesis in RAGE signal transduction. Phosphorylation of p38 (p-p38) promotes cell inflammation. In the present study, we observed that MPTP exposure increased p-p38 significantly, while cotreatment of MPTP with RAGE-siRNA attenuated the upregulation of p-p38/p-38 ratio. In the RAGE-NC and RAGE-siRNA group, p-p38 expression was not significantly changed compared to the control group. This indicates that p38 MAPK may be involved in the RAGE signal transduction and RAGE-siRNA exerts beneficial roles against MPTP toxicity (Figure 6).

## Discussion

Neuroinflammation, which is characterized by activated microglia, infiltrating T cells, and subsequent sustained release of inflammatory mediators at sites of neuronal injury, is a prominent contributor to the pathogenesis of neurodegenerative diseases including PD (Chen et al., 2003, 2005, 2008; Hirsch and Hunot, 2009; Qian et al., 2010; Hill-Burns et al., 2011). The first evidence for neuroinflammatory processes in PD was from serum or cerebrospinal fluid and post-mortem studies. Activated microglial cells and a 30% increase of astroglial cell density were observed within the SN of patients with PD at post-mortem (McGeer et al., 1988; Damier et al., 1993; Mirza et al., 2000; Imamura et al., 2003). The density of CD8 + and CD4 + T cells in the brains of patients with PD were higher than that in brains in healthy individuals (Brochard et al., 2009). Further, the presence of neuroinflammatory processes at postmortem has also been confirmed on a molecular basis. Levels of proinflammatory mediators, including TNFα, IL-1β, IL-6, and reactive oxygen species were elevated in the brains and peripheral blood mononuclear cells of PD patients (McGeer et al., 1988; Nagatsu et al., 2000; Reale et al., 2009a, b). Moreover, in animal models of the disease, cellular and molecular events associated with neuroinflammation were also reproduced with intracerebral 6-hydroxydopamine injection or peripheral injection MPTP or rotenone (Hirsch and Hunot, 2009). Thus, inflammation may be the fundamental process contributing to neuron death in PD and anti-inflammation might be a new promising target for neuroprotective therapy for PD.

RAGE is a new member of the immunoglobulin superfamily, which was first identified and described in 1992 as a RAGE (Neeper et al., 1992). RAGE was primarily expressed in neurons, microglia, and vascular endothelial cells in the central nervous system (Kierdorf and Fritz, 2013). Recently, it has been proposed that RAGE played a crucial role in neuroinflammation and neurodegeneration. In AAD, binding of AGEs and Aβ to RAGE was reported to stimulate activation of transcription factor NF-κB, which, in turn, induced the release of various cytokines. Moreover, RAGE ligation stimulated the generation of reactive oxygen species, which was implicated in the early toxic events that result in progression of AD (Chuah et al., 2013; Ray et al., 2016). Regarding PD, RAGE was also speculated as a contributor to inflammation of PD.

As one of the RAGE ligand, AGEs resulting from glycation of α-synuclein were first found in SN and locus coeruleus of peripheral Lewy bodies (Padmaraju et al., 2011). Later, an increased RAGE protein and messenger RNA levels had been investigated in PD patients and animal models (Dalfo et al., 2005; Sathe et al., 2012). To date, increasing reports demonstrated that RAGE activation induced a series of signal transduction cascades and led to the activation of transcription factor NF-κB as well as increased expression of cytokines including COX2, TNFα, and IL1 (Bianchi et al., 2011; Sathe et al., 2012). *In vivo* evidence showed that S100B was increased in PD and ablation protected against MPTP-induced toxicity through the RAGE and TNF-α pathway (Sathe et al., 2012). The administration of neutralizing antibodies to HMGB1 suppressed MPTP-induced HMGB1 and RAGE upregulation while attenuating MPTP-induced dopaminergic cell death in a dose-dependent manner (Santoro et al., 2016).

In this study, we showed an upregulation of RAGE in the SN of mice treated with the neurotoxin MPTP and RAGE ablation protected against MPTP-induced deficit of DA, DOPAC, and HVA content in the striatum and loss of TH neurons in the SN. RAGE suppression also improved coordination and balance of mice. Then, in our study, we demonstrated that RAGE activation was accompanied by an upregulation of COX2. The enzyme cyclooxygenase-2, expressed in several cell types in response to growth factors, cytokines, and pro-inflammatory molecules, are instrumental in PD neurodegeneration (Minghetti, 2004; Teismann, 2012). In both MPTP mice and human PD samples, it was shown an upregulation of COX-2 in the nigrostriatal dopaminergic neurons (Knott et al., 2000; Teismann et al., 2003). Evidence showed that COX-2 participated in the MPTP neurotoxic process and affected dopaminergic neurons in the SN and nerve fibers in the striatum (Teismann et al., 2003). But this neurodegeneration was mitigated in COX-2 deficient mice, which further supported the involvement of COX-2 in PD neurodegeneration (Feng et al., 2002). COX-2 toxicity is presumably mediated by increasing the levels of prostanoids. Accordingly, the release of prostanoids-2 by COX-2-positive neurons promoted the production of microglial-derived mediators, which, in turn, helped in killing neurons (Teismann et al., 2003). Up to now, mounting evidence suggested that elevated levels of COX2 were ultimately deleterious to dopaminergic neurons. However, the regulation of COX2 expression by ligation of RAGE is still controversial and the related molecular signaling events remain unclear.

It has been well documented that NF-κB signaling pathways were involved in the RAGE activation (Lin et al., 2009). Engagement of RAGE resulted in the cascade signaling and eventually led to the activation of NF-κB, which is the key transcriptional regulator in the inflammation process (Lin et al., 2009). Normally, NF-κB resided in the cytoplasm sequestered by the inhibitor molecule IκBα. Upon activation, IκBα was rapidly phosphorylated and degraded, resulting in the dissociation and translocation of NF-κB (preferentially the NF-κB p65 subunit) into the nucleus. Subsequent to nuclear translocation, p65 subunit bound to DNA sequences and initiated transcription of inflammatory response-related mRNAs, such as COX-2, TNFα, and IL-6, and thereby ultimately augmented their synthesis and secretion (Bierhaus et al., 2005; Xie et al., 2013). Interestingly, the activation of the RAGE-NF-κB signaling also resulted in an upregulation of RAGE itself, which amplified the initial signal and further enhanced inflammation (Salahuddin et al., 2014). This was supported by the fact that RAGE gene contained functional NF-κB binding sites in its proximal promoter and has been shown to be a direct target gene for NF-κB signaling (Li and Schmidt, 1997; Kierdorf and Fritz, 2013; Tobon-Velasco et al., 2014). It was also reported that NF-κB signaling pathways was involved in COX2 expression (Lin et al., 2009). Bianchi et al. (2010) revealed that direct inhibition of NF-κB resulted in negation of S100B/RAGE-induced upregulation of COX-2 expression. Selective inhibition of NF-κB by the use of NF-κB essential modifier-binding domain peptides was shown to prevent dopaminergic neuronal loss in a mouse model of PD, by preventing the expression of proinflammatory molecules (Ghosh et al., 2007). In the present study, MPTP-induced NF-κB activation was investigated by immunoblotting. Our data clearly demonstrated that the MPTP-induced IκB phosphorylation was effectively inhibited by RAGE siRNA treatment. These results indicated that NF-κB activation was involved in the RAGE signal pathway and contributed to the inflammation.

The stimulation of RAGE was able to activate the MAPK signaling cascade (Lander et al., 1997; Xie et al., 2013). MAPK signal transduction was mainly composed of Jun-N-terminal kinase (JNK), p38, and extracellular signal-regulated kinases (ERK) signaling pathways, which were required for the induction of NF-κB activation as upstream effectors (Taguchi et al., 2000). Among these, p38MAPK signal pathway caught our attention because of its important role in the activation of NF-κB. p38 was known to phosphorylate IκBα, leading to its dissociation from the p65 subunit, thus facilitating the translocation of p65 to the nucleus (Karunakaran and Ravindranath, 2009). Gao et al. (2013) reported that BV2 cells (a mouse microglial cell line) treated with rotenone were induced to release inflammatory cytokines via NF-κB signaling pathway, which was strictly dependent on p38 MAPK. Animal trials also revealed that nuclear translocation of NF-κB in MPTP-treated mice was mediated by activation of p38MAPK in the SN (Karunakaran and Ravindranath, 2009). In the present study, our data showed that MPTP administration produced a strong activation of p38MAPK, which correlated with the increase of NF-κB and inflammatory mediators. Moreover, RAGE ablation treatment resulted in a significant inhibition on the phosphorylation p38 along with reduced production of pro-inflammatory cytokines. Thus, we speculated that RAGE-dependent p38 MAPK activation might contribute to upregulate COX-2 expression via NF-κB, which was analogous to the case of chondrocytes and monocytes. A previous study conducted in human chondrocytes showed that endoplasmic reticulum stress and the expression of COX2 induced by AGEs was through eIF2α, p38-MAPK, and NF-κB pathways (Rasheed and Haqqi, 2012). Additionally, regulation of COX2 expression in monocytes by ligation of RAGE was also regulated by p38 and NF-K B pathways (Shanmugam et al., 2003). However, in murine BV-2 microglial cell line, direct blockade of NF-κB resulted in an evident inhibition of COX-2 expression, but inhibition of the p38 MAPK did not affect S100B/RAGE-induced upregulation of COX-2 expression (Bianchi et al., 2010). This discrepancy might be explained by the observation that other possible RAGE-dependent NF-κB-activating kinases could also activate NF-κB and trigger the COX2 expression.

Taken together, we demonstrated that RAGE had been involved into a proinflammatory change in the process of PD within animal models. Results also indicated that p38 MAPK activation mediated RAGE-NF-κB-dependent secretion of proinflammatory cytokines, thereby inducing DA degradation in the striatum and TH neuron loss in the SN. In this paper, we used the siRNA to silence RAGE expression by injection of the lentivirus into SN for the first time. By RAGE ablation, NF-κB translocation to nuclear was decreased, accompanied by downregulation of COX2 expression, one of the proinflammatory cytokines. Our study has implications for the pathogenesis of PD and indicates that delivery of RAGE inhibition may represent new appealing approaches for therapy.

## Data Availability Statement

The raw data supporting the conclusions of this manuscript will be made available by the authors, without undue reservation, to any qualified researcher.

## Ethics Statement

The animal study was reviewed and approved by the Committee on the Ethics of Animal Experiments of Qingdao University.

## Author Contributions

AX and CZ conceived the experiments, read, and revised the manuscript. XW and XS wrote the manuscript, designed, and performed the experiments. XZ, MN, and JW performed data extraction. All authors read and approved the final manuscript.

## Conflict of Interest

The authors declare that the research was conducted in the absence of any commercial or financial relationships that could be construed as a potential conflict of interest.
